# Effect of minimally invasive non-surgical periodontal therapy in former smokers with periodontitis on salivary IL-1β and PGE-2 profile

**DOI:** 10.3389/froh.2026.1779330

**Published:** 2026-03-05

**Authors:** Noura Mohamed Elshiaty, Saad Mohammad Al-Zubaidi, Fatma Hamed El Demerdash, Hussien Elkholy, Ahmed Youssef Gamal

**Affiliations:** 1 Faculty of Dentistry, Ain Shams University, Cairo, Egypt; 2 College of Dentistry, University of Ha’il, Ha’il, Saudi Arabia; 3 Faculty of Dentistry, Misr International University, Cairo, Egypt; 4 Institute of Psychiatry, Ain Shams University, Cairo, Egypt; 5 Faculty of Dentistry, Misr University for Science and Technology, Cairo, Egypt

**Keywords:** health behavior, inflammatory mediators, minimally invasive non-surgical therapy, periodontal diseases, periodontitis, public health, smoking, smoking cessation

## Abstract

**Objectives:**

Limited data are available regarding salivary inflammatory marker levels following conventional versus minimally invasive non-surgical periodontal therapy and smoking cessation. This study aimed to evaluate the effects of conventional non-surgical periodontal therapy (CNST) versus minimally invasive non-surgical periodontal therapy (MINST) on the levels of salivary inflammatory markers, specifically interleukin-1β (IL-1β) and prostaglandin E2 (PGE2), in patients with stage III grade A/B periodontitis. Moreover, it investigated the impact of smoking cessation on these biomarkers and compared outcomes among smokers, non-smokers (NS), and individuals who quit smoking.

**Methods:**

A randomized controlled biochemical trial was conducted with 40 patients divided into four groups: smokers who quit and received conventional therapy (SQ1), smokers who quit and received minimally invasive therapy (SQ2), smokers continuing to smoke (SC), and NS.

**Results:**

Both the SQ2 and NS groups reported significant reductions in salivary PGE2 and IL-1β levels at both observation periods compared with the SC group.

**Conclusions:**

MINST appeared more effective in controlling inflammatory markers among patients who quit smoking compared with conventional non-surgical therapy.

## Introduction

Smoking is one of the most significant risk factors in the development and progression of periodontal disease (1). Smoking has been confirmed as a risk factor that enhances the progression of periodontitis and reduces response to therapy in the new periodontitis classification proposed by the 2018 World Workshop on the Classification of Periodontal and Peri-Implant Diseases and Conditions (2). It has been demonstrated that cigarette smoking negatively affects clinical outcomes following both surgical and non-surgical periodontal therapy (3) and during periodontal supportive therapy following active therapy (4). Following regenerative therapy, compromised healing of intrabony and gingival recession defects in smokers has also been reported (5). In addition, cigarette smoking may directly affect the self-healing potentials of the alveolar process even in the absence of plaque biofilm (6). Cigarette smoking has been shown to impair periodontal healing by negatively affecting fibroblast function, reducing vascularity, and altering immune response, thereby compromising regenerative potential (5, 7). Nicotine exposure, along with reduced oxygen transport and metabolism caused by carbon monoxide (CO), induces reduced peripheral blood supply because of vasoconstriction (8). Therefore, it appears that smoking interferes with multiple stages of the reparative and regenerative processes in periodontal wounds, thereby compromising overall healing (9).

IL-1β is reported to play an integral role in the etiology of periodontal disease (10). Similarly, PGE2 has been found in tissue samples of patients with gingivitis and is believed to be involved in the pathogenesis of periodontal disease (11). Salivary IL1-β and PGE2 levels have been reported to increase with periodontal disease severity and decrease with therapy, suggesting their utility as surrogate indicators of periodontal disease presence and severity (12). IL-1β concentrations have been proven to be higher in smokers than in healthy subjects and further elevated in smokers with periodontitis compared with non-smokers (NS) with periodontitis, emphasizing the interaction between salivary IL-1β and smoking (13).

Smoking cessation is an influential factor in periodontal therapy, and smokers should be encouraged to stop smoking as a part of their periodontal management (14). Cessation of smoking was reported to significantly reduce probing depths after non-surgical treatment over a 12-month period (15). It has been shown that the rate of tooth loss among men who left smoking was about 50% lower than the rate of current smokers (16). Smoking cessation was reported to decrease periodontitis development as well as to enhance treatment response. On the other hand, studies reported that the effect of smoking as a risk factor for periodontitis could persist for up to 10 years after quitting (17).

Evidence reporting the effect of smoking cessation and minimally invasive non-surgical periodontal therapy (MINST) on inflammatory biomarkers and periodontal health is very limited. Addressing this gap could help to shed light on the most effective approach to reverse elevated inflammatory mediator levels associated with smoking. The primary aim of this study was to evaluate the effect of 3 months of smoking cessation on inflammatory markers—IL-1β and PGE2—following MINST compared with CNST in patients with stage III grade A or B periodontitis. In order to monitor periodontal status during the follow-up period and assess patient compliance, periodontal soft tissue parameters were evaluated throughout the 3-month biochemical study period. To the best of our knowledge, this is the first study to evaluate the effect of MINST and smoking cessation on biochemical parameters in patients with stage III grade A and B periodontitis.

## Materials and methods

### Patient selection and grouping

Inclusion criteria comprised patients with stage III grade A/B periodontitis, defined as CAL ≥5 mm and PD ≥6 mm. Patients were randomized into four groups (*n* = 10/group): smokers willing to quit receiving CNST (SQ1), smokers willing to quit receiving MINST (SQ2), current smokers receiving CNST (SC), and non-smokers receiving CNST (NS). Ethical approval (FDASU-RecM121410) and informed consent were obtained.

### Details of patient selection and grouping

A total of 40 systemically healthy patients of both sexes, in an age range from 30 to 50 years, were selected from the outpatient clinic of the Department of Periodontology, Faculty of Dentistry, Ain Shams University, between April 2020 and March 2021. Patients were included if they had stage III grade A or B periodontitis (2), with clinical attachment loss (CAL) ≥5 mm, pocket depth (PD) ≥6 mm, and evidence of interproximal radiographic bone loss in a form of two- or three-wall intrabony defect depth of ≥3 mm, as detected by cone-beam computed tomography (CBCT). Each patient contributed one interproximal intrabony defect of a molar or premolar tooth. Smokers were defined as those smoking 10 cigarettes/day. Those willing to quit smoking (20 subjects) were divided randomly into two groups. The first group (SQ1) consisted of 10 subjects receiving CNST. The second group (SQ2) also included 10 subjects receiving MINST. Patients unwilling to quit smoking and who continued to smoke were included in the third group (SC), comprising 10 subjects treated with CNST. The fourth group (NS) included 10 non-smokers, who also received CNST. Exclusion criteria included patients with any systemic illness known to affect the outcome, patients undergoing systemic antimicrobial or anti-inflammatory drug therapy within the last 3 months prior to the study, patients unable to perform routine oral hygiene procedures, patients using other forms of tobacco, and pregnant women. This study was reviewed by the Ethical Committee of the Faculty of Dentistry, Ain Shams University (FDASU-RecM121410), and registered under identifier: NCT06145685. The proposed nature of the study was explained to all participants, and written informed consent was obtained from each patient.

### Sample size calculation

A power analysis was performed to ensure appropriate power for testing of the null hypothesis that there is no difference in mediator levels between different study samples. By adopting an alpha level of 0.05 and a beta of 0.2, i.e., power = 80%, and an effect size (f) of (0.479) calculated from the results of a prior study (18), the predicted sample size (*n*) was a total of 40 samples (i.e., 10 samples per group). Sample size calculation was performed using G*Power version 3.1.9.7.

### Intervention

All patients underwent full-mouth scaling and root debridement. The MINST protocol employed piezo-electric devices and mini-curettes to minimize soft tissue trauma, whereas the CNST protocol followed standard ultrasonic instrumentation.

All individuals were subjected to thorough extra-oral and intraoral examinations, and any deviations from normality were recorded. Periodontal evaluation was performed using the gingival index (19), plaque index (20), PD measure with William's graduated periodontal probe from the base of the pocket to the free gingival margin at the most affected site, and CAL measured from the base of the pocket to the cemento-enamel junction (21). Patients' detailed tobacco use history was taken from the smokers' groups to determine the degree of dependency, including smoking brand, number of cigarettes smoked per day, and duration of smoking habit (22).

Smokers were approached using the Brief Intervention technique (Ask, Advise, Assess, Assist, and Follow-up) in order to motivate them and assess their readiness to quit (23). Those who expressed their willingness to quit were referred to the Tobacco Dependence Treatment (TDT) clinic at the institute of Psychiatry, Ain Shams University, as part of cross-sectional collaboration agreement with the Department of Periodontology. These patients received counseling in the form of 4–8 weekly sessions by a specialized physician (H.A.E), without prescribing any medication. The counseling package included conversations on smoking habits, diversion strategies from smoking, withdrawal, and benefits of cessation. In addition, smoking cessation motivation was reinforced at every dental appointment. To verify smoking cessation status, participants in the smoking quitters group were monitored using exhaled CO levels and salivary cotinine testing. CO levels were measured at baseline and during follow-up visits (e.g., 1 and 3 months after intervention) using a handheld CO monitor. A CO reading of <6 parts per million (ppm) was considered indicative of successful smoking cessation (24). Moreover, salivary cotinine levels were measured at the 1- and 3-month follow-ups to provide biochemical verification of sustained abstinence. Cotinine testing was performed using enzyme-linked immunosorbent assay (ELISA) kits, with cotinine levels below the threshold of detection indicating non-smoking status (25). This combined approach ensured both short-term and long-term verification of smoking abstinence, enhancing the reliability of the cessation data. Smokers unwilling to stop smoking were enrolled into the third group.

Randomization of the smoking quitters group was carried out using a computerized random number generator (Random.org; https://www.random.org). The allocation sequence was produced using software and preserved in invisibly sealed envelopes. All patients received cause-related periodontal treatment by the same blinded operator (NE), which included oral hygiene instructions, supra- and subgingival scaling, and root debridement under local anesthesia without any adjuncts (antimicrobial or host-modulating agents). Full-mouth subgingival instrumentation was performed using hand instruments and ultrasonic scalers, followed by teeth polishing with rubber cups under 2.5× magnification loupes. This was performed according to the protocol of MINST (26) for SQ2 patients. MINST began with thorough root surface debridement up to the deepest part of the periodontal pocket, guided by contentious bone sounding with a periodontal probe. Efforts were made to decrease trauma to the soft tissues by using piezo-electric devices with miniature tips (Woodpecker P3 tip, Woodpecker, Guilin, China), followed by Gracey mini-curettes, including a set of “after five” and “micro mini five” curettes (Hu-Friedy, Chicago, IL, USA). During manipulation, instruments were inserted lightly through the periodontal pocket in order to maintain the soft tissues stability. Root surface smoothing and subgingival curettage were avoided. Attempts were made to maintain a stable blood clot within the intrabony defect following debridement by avoiding subgingival irrigation. Full-mouth conventional non-surgical therapy was performed for the other groups following the same sequence of MINST, except that conventional ultrasonic and hand instruments were used for subgingival instrumentation.

All patients were enrolled in a supportive therapy protocol including monthly oral hygiene instructions and maintenance supra- and subgingival debridement. Baseline salivary samples were obtained from all patients for IL1B and PGE2 evaluation at the first visit, before any intervention by a blinded assessor. Samples were collected again from all subjects at 1 and 3 months after the baseline sample. Soft tissue clinical parameters were evaluated by a blinded assessor at 1- and 3-month follow-up visits to confirm patient compliance and monitor disease activity.

### Salivary sample collection and processing

Whole unstimulated saliva (WUS) was collected from all participants before treatment, and at 1 and 3 months following treatment. Samples were analyzed for IL-1β and PGE2 using ELISA kits. Collection of WUS followed standard techniques, as described by Navazesh (27). Briefly, subjects refrained from eating or drinking for at least 1.5 h prior to evaluation. Samples were obtained by requesting subjects to swallow first and then tilt their head forward, and saliva was collected by careful aspiration using a sterile syringe. All samples were immediately stored at −20°C until assayed. All salivary samples were kept in a refrigerator at – 20 in order to stop or significantly slow down bacterial growth and enzymatic activity, which would otherwise alter the composition of the sample. Samples were disposed of according to the laboratory instructions. IL-1β and PGE2 concentrations were measured using a commercially available ELISA kit supplied by Quantikine (R&D Systems, McKinley Place, USA). The resultant concentrations were multiplied by the dilution factor to get the final concentration.

### Statistical analysis

Data were analyzed using SPSS, version 25. A *p*-value of ≤0.05 was considered significant. Statistical analysis for clinical data was analyzed by applying the paired *T*-test for intragroup comparison between the two time intervals, and one-way ANOVA followed by the Tukey *post hoc* test for intergroup comparison between the different groups. For biochemical data, one-way ANOVA followed by Tukey *post hoc* test was applied for intra- and intergroup comparison. A *p*-value of ≤0.05 was considered statistically significant (95% significance level), while a *p*-value of ≤0.001 was considered highly statistically significant (99% significance level). The Shapiro–Wilk test was used to assess the normality of the data. The negative value of the percentage change indicated that the baseline value changed to a higher value after time *t*, while the positive value of the percentage change meant that the baseline value changed to a lower value after time *t*. Statistical evaluation was performed using the SPSS statistical package (version 25, IBM Co., USA).

## Results

### Key findings

Significant reductions in PGE2 and IL-1β levels were observed in the SQ2 and NS groups at both observation periods compared with the SC group.

The SQ2 group achieved comparable biomarker levels to the NS group after 3 months, indicating the combined effectiveness of smoking cessation and MINST.

The SQ2 group achieved the highest percentage reduction in probing depth (50.57%) compared with the SC group (31.43%).

**Figure 1:**
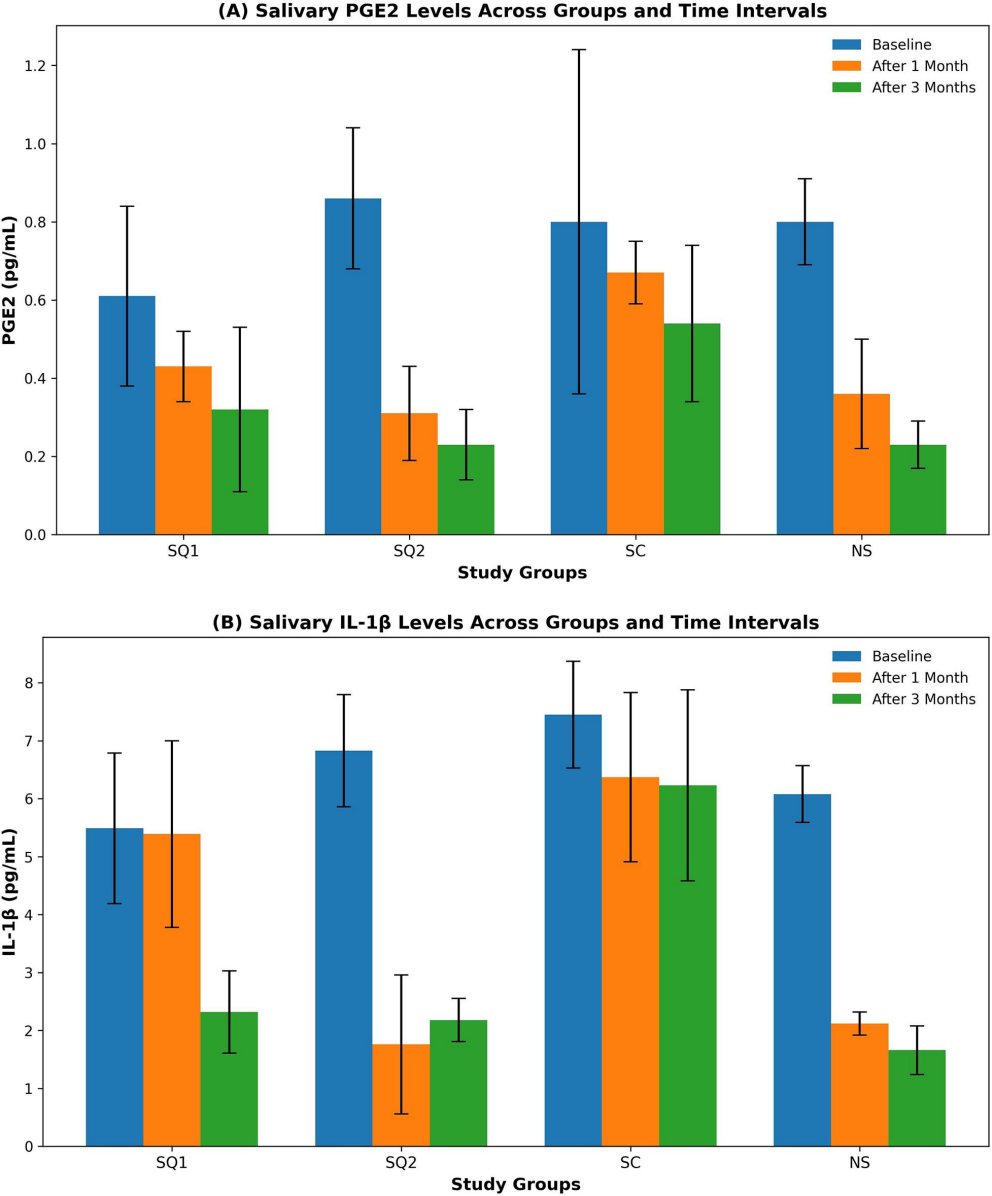
Bar chart representing the mean and SD of biomarkers (PGE2 and IL-1β) across groups at different intervals. PGE2 and IL1β levels measured across four groups (*n* = 10/group) at three time intervals (baseline, after 1 month, and after 3 months). The top panel represents PGE2 levels (pg/mL) and the bottom panel represents IL1β levels (pg/mL). The groups are defined as follows: smokers who quit and received CNST (SQ1), smokers who quit and received MINST (SQ2), current smokers receiving CNST (SC), and non-smokers receiving CNST (NS). Error bars indicate standard deviation. Color codes represent the time intervals: orange for baseline, yellow for after 1 month, and crimson for after 3 months.

Tables 1, 2 and Figure 1 illustrate statistical differences among groups.

**Table 1:** Mean values and statistical differences of PGE2 and IL-1β levels among groups.

		Baseline	After 1 month	After 3 months	*P*-value*
PGE2	SQ1	0.61 ± 0.23^Aa^	0.43 ± 0.09^Bab^	0.32 ± 0.21^ABb^	0.031^S^
	SQ2	0.86 ± 0.18^Aa^	0.31 ± 0.12^Bb^	0.23 ± 0.09^Bb^	0.001^HS^
	CS	0.8 ± 0.44^Aa^	0.67 ± 0.08^Aa^	0.54 ± 0.2^Aa^	0.123^NS^
	NS	0.8 ± 0.11^Aa^	0.36 ± 0.14^Bb^	0.23 ± 0.06^Bb^	0.003^S^
	*P*-value**	0.530^NS^	0.003^S^	0.028^S^	
IL1β	SQ1	5.49 ± 1.3^Ba^	5.39 ± 1.61^Aa^	2.32 ± 0.71^Bb^	0.003^S^
	SQ2	6.83 ± 0.97^ABa^	1.76 ± 1.2^Bb^	2.18 ± 0.37^Bb^	<0.001^HS^
	CS	7.45 ± 0.92^Aa^	6.37 ± 1.46^Aa^	6.23 ± 1.65^Aa^	0.346^NS^
	NS	6.08 ± 0.49^ABa^	2.12 ± 0.2^Bb^	1.66 ± 0.42^Bb^	<0.001^HS^
	*P*-value**	0.031^S^	<0.001^HS^	<0.001^HS^	

Small and capital letters for pairwise comparison between different time intervals and different groups, respectively (Tukey *post hoc* test), and the means with different superscripts are statistically significant. S, statistically significant at *P* ≤ 0.05; NS, non-significant *P* < 0.05; HS, highly significant at *P* ≤ 0.001.

* *P*-value for intragroup comparison between the three time intervals (ANOVA).

** Overall *P*-value for intergroup comparison between the four groups (ANOVA test).

**Table 2:** Clinical parameters (GI, PI, CAL, and PD) comparison before and after treatment.

		Baseline	After 3 months	Percentage of change	*P*-value*
GI	SQ1	2 ± 0.71**^A^**	0.75 ± 0.5**^A^**	66.67 ± 23.57**^A^**	**0** **.** **004^S^**
	SQ2	2.2 ± 0.45**^A^**	0.8 ± 0.45**^A^**	63.33 ± 21.73**^A^**	**0** **.** **005^S^**
	CS	2.2 ± 0.45**^A^**	0.6 ± 0.55**^A^**	73.33 ± 25.28**^A^**	**0** **.** **003^S^**
	NS	2.2 ± 0.45**^A^**	0.4 ± 0.55**^A^**	83.33 ± 23.57**^A^**	**0.001^HS^**
	*P*-value**	0.907**^NS^**	**0.627^NS^**	**0.575^NS^**	
PI	SQ1	2.2 ± 0.45**^A^**	0.5 ± 0.58**^A^**	79.17 ± 25**^A^**	**0** **.** **006^S^**
	SQ2	2.2 ± 0.45**^A^**	0.8 ± 0.45**^A^**	63.33 ± 21.73**^A^**	**0** **.** **009^S^**
	CS	2 ± 0.71**^A^**	0.8 ± 0.45**^A^**	50 ± 35.36**^A^**	**0** **.** **012^S^**
	NS	2 ± 0**^A^**	0.8 ± 0.45**^A^**	60 ± 22.36**^A^**	**0** **.** **007^S^**
	*P*-value**	**0.828^NS^**	**0.743^NS^**	**0.467^NS^**	
CAL	SQ1	4.8 ± 0.45**^A^**	2.75 ± 0.5**^A^**	41.25 ± 14.36**^A^**	**0** **.** **016^S^**
	SQ2	4.4 ± 0.89**^A^**	2.2 ± 0.45**^A^**	46 ± 26.08**^A^**	**0** **.** **020^S^**
	CS	4.4 ± 0.55**^A^**	2.8 ± 0.45**^A^**	35 ± 15.41**^A^**	**0** **.** **016^S^**
	NS	4 ± 0.71**^A^**	2.6 ± 0.55**^A^**	32 ± 23.61**^A^**	**0** **.** **026^S^**
	*P*-value**	**0.347^NS^**	**0.249^NS^**	**0.722^NS^**	
PD	SQ1	6.6 ± 1.14**^A^**	3.75 ± 0.5**^AB^**	45.83 ± 10.17**^AB^**	**0** **.** **007^S^**
	SQ2	6 ± 0.71**^A^**	3 ± 0.71**^B^**	50.57 ± 6.11**^A^**	**0** **.** **002^S^**
	CS	6.8 ± 0.84**^A^**	4.6 ± 0.55**^A^**	31.43 ± 12.07**^B^**	**0** **.** **011^S^**
	NS	6.4 ± 0.55**^A^**	3.4 ± 0.89**^AB^**	46.67 ± 14.83**^AB^**	**0** **.** **023^S^**
	*P*-value**	**0.495^NS^**	**0.015^S^**	**0.041^S^**	

Capital letters for pairwise comparison between different groups (Tukey *post hoc* test) and the means with different superscripts are statistically significant different at *P* ≤ 0.05. S, statistically significant at *P* ≤ 0.05; NS, non-significant *P* < 0.05; HS, highly significant at *P* ≤ 0.001.

The bold values indicate statistically significant differences at *P* ≤ 0.05 between groups (Tukey *post hoc* test).

* *P*-value for intragroup comparison between the two time intervals (Paired *T*-test).

** Overall *P*-value for intergroup comparison between the four groups (ANOVA test).

All patients completed the study with no dropouts. Patients in the SC, SQ1, and SQ2 groups reported smoking 10 cigarettes or less per day of the same brand during the last 5 years. The SQ1 group included 10 patients— nine men and one woman, with a mean age of 27.9 years—who successfully quit smoking following the smoking cessation program and received CNST. The SQ2 group included 10 men, with a mean age of 28.3 years, who also successfully quit smoking following the smoking cessation program and received MINST. The mean time period for complete smoking cessation was 5.3 week. The SC group consisted of 10 men, with a mean age of 28.6 years, who refused to quit smoking and received CNST. The NS group included 10 male non-smokers, with a mean age 32.4 years, who received CNST. No significant differences were reported for baseline demographic characteristics between all groups (*P* > 0.05).

Table 1 presents the mean values of PGE2 and IL-1β in PG/mL and the statistical differences between groups at baseline and the two observation periods. For PGE2 biomarkers, the lowest mean of PGE2 was achieved after 3 months in the SQ2 and NS groups, while the highest mean was achieved at baseline in the SQ2 group. For IL1β biomarkers, the lowest mean of IL1β was achieved after 2 months in the NS group, while the highest mean was achieved at baseline in the current smokers (CS) group. At baseline, there was no significant difference in salivary levels of PGE2 or IL-1β among the groups. There was a gradual significant reduction in the mean values of PGE2 and IL-1β in all groups during the two observation periods, except for the SC group. The SC group reported the highest levels of PGE2 and IL-1β, and in spite of its gradual reduction, the values remained non-significant from baseline during different observation periods. The SQ2, SQ1, and NS groups showed significant reduction in both PGE2 and IL-1β levels throughout the two observation periods compared to the SC group. No significant differences were observed between the SQ2 and NS groups during the two observation periods for both PGE2 and IL-1β. At both observation periods, the SQ2 group showed significantly lower PGE2 and IL-1β levels than the SQ1 group (Tables 1, 2, Figure 1).

For intergroup comparisons, the results of Tukey *post hoc* test indicated that there were no significant differences between the four groups at baseline for all parameters (PD, CAL, IBC, PI (plaque index), and GI (gingival index)). After 3 months, the same results were achieved for all parameters, except for PD. At this point, there was a significant difference between the CS group and the SQ2 group. According to the ANOVA test, the overall *p*-value for the intergroup comparison was not statistically significant for all parameters at the two time intervals, except for PD after 3 months. For PD after 3 months, there was a slightly significant difference between the groups, and the overall *p*-value was significant at the 0.05 level. Moreover, the results of the comparison of the change percentage across the four groups gave the same results.

### Statistical overview

Tables and figures present detailed comparisons of biomarker levels and clinical parameters among the groups (Tables 1, 2, Figure 1). The SQ2 group achieved comparable biomarker levels to the NS group after 3 months, indicating the combined effectiveness of smoking cessation and MINST.

## Discussion

Smoking has consistently been associated with more severe periodontitis and less predictable outcomes compared with never-smokers (28). Periodontal therapies have been reported to be less effective in 25% of the population with moderate and severe periodontitis (29). Abnormal cytokine expression from smoking accelerates periodontitis development and reduces resolution after therapy (30). Smoking cessation protocols have been reported to reduce smoking-related oral effects and represent a great preventive measure for many systemic diseases (31). It has been suggested that smoking cessation initiatives could help to prevent a great percentage of new periodontitis cases across populations (32). Despite extensive evidence on the negative effects of smoking on periodontal health and the positive effects of smoking cessation on periodontitis development, it appears that few studies have evaluated the effects of smoking cessation on salivary inflammatory markers—the objective of the present study.

Smoking has been shown to induce an elevated expression of IL-1β, which promotes tissue destruction, bone resorption, and production of matrix metalloproteinases and prostaglandin E2, leading to the destruction of periodontal tissue (33). The present study employed MINST to determine if it could contribute to controlling inflammatory mediators following smoking cessation compared with conventional non-surgical therapy. Nibali et al. (34) were the first to evaluate intrabony defect fill following MINST. They reported that long junctional epithelium following minimally invasive non-surgical periodontal therapy may be accompanied by intrabony defect fill or increased bone density. Minimally invasive non-surgical therapy was reported to have similar positive clinical effects as minimally invasive surgical techniques with or without the additional use of biologics (35). To our knowledge, this is the first study to evaluate biological outcomes in smokers who quit the habit and receive MINST. Previous clinical trials have only reported outcomes in separate cohorts of smokers, ex-smokers, and non-smokers following non-surgical treatment.

Short-term evaluation of inflammatory biomarkers in the present study was performed to determine whether a 3-month smoking cessation period could reduce systemic and local inflammation prior to potential surgical intervention. Rodrigues et al. reported that the concentrations of inflammatory markers (TNF-α, IL-6, IL-8, and IL-10) in blood serum were significantly decreased after 30 days of smoking abstinence when compared to active smokers. They concluded that short-term smoking abstinence decreased systemic inflammation. Other studies have also reported significant reductions in serum cotinine after 12 weeks of smoking cessation. The biochemical method of assessment used in the present study measured salivary IL1β and PGE-2 biomarkers. Saliva was chosen due to its convenience and non-invasive sample collection, compared to blood and gingival crevicular fluid. Saliva contains various biologic ingredients and bacteria, which can be used to evaluate oral health status (36). The high sensitivity and specificity of salivary IL1β and PGE2 in identifying periodontitis further support their potential as biomarkers for diagnosis of periodontitis presence and severity (12). IL-1β plays a significant role in the innate immune response by inducing the synthesis and secretion of other mediators that contribute to inflammatory changes and tissue damage. IL-1β activates the synthesis of nitrous oxide (10). Prostaglandin E2 accelerates vascular changes responsible for inflammation and increases blood flow to the infection site (37). A 12-week smoking cessation program was reported to reduce circulating concentrations of ET-1 and TNF-α for at least a year (38). In the present study, the control group (SC) consisted of smokers who continued smoking and received conventional non-surgical therapy.

At baseline all groups showed significantly high levels of mediators compared with the 1- and 3-month observation periods. This could be attributed to the nature of the selected cases with deep pocket depth and high levels of gingival index scores before therapy in all groups. Patients with bleeding on probing and deep pocket depths had higher levels of GCF IL-1β (12). At baseline, there was no significant difference in salivary levels of PGE2 or IL-1β across the groups. Studies have found that there is no significant difference in the level of cytokines between active periodontitis patients with and without smoking (39). Gradual significant reduction in the mean values of PGE2 and IL1β in the SQ1, NS, and SQ2 groups following both conventional and minimally invasive non-surgical therapy was reported in this study during the different observation periods. At 3 months, no significant differences among these three groups were reported for IL1 β, a finding which could reflect the positive effect of the short-term smoking cessation on the local mediator levels, approaching non-smoker levels. On the other hand, the SC group maintained a significantly high baseline value for up to 3 months compared to the other groups. This finding could be an indicator that conventional non-surgical therapy is not sufficient to control elevated inflammatory mediator levels in smoker' with periodontitis. A recent systematic review reported that after periodontal therapy, smokers with periodontitis showed significantly higher IL-1β levels in their GCF than non-smoking patients (39). The SQ2 and NS groups reported significantly higher levels of PGE2 reduction compared to the SQ1 and SC groups. This could be attributed to the double effect of smoking cessation and the enhanced efficacy of minimally invasive inserts, which allow deeper access into diseased areas (40). The use of mini-inserts without any time limitations also contributed to the positive effects. This finding may suggest that smoking cessation combined with minimally invasive therapy enhances the reduction of inflammatory burden more effectively than conventional therapy alone.

Soft tissue clinical parameters reported marked reduction of both GI and PI at the 1- and 3-month observation periods compared with the baseline (Table 2). Pocket depth and attachment levels were gently checked for disease activity and reported significant reduction. These findings, combined with biochemical results, parallel those of Lee et al. (41) who reported that pro-inflammatory cytokine IL-1β levels are inversely correlated with the recovery rate of periodontitis clinical parameters following non-surgical treatment. The use of MINST was reported in many studies to be a reliable option for the treatment of intrabony defects (34, 42). Nibali et al. (34) reported prolonged treatment outcomes following the use of MINST for intrabony lesions. The greater reduction of inflammatory mediators observed in the SQ2 group may reflect the synergistic effect of smoking cessation and minimally invasive instrumentation.

Within the limitations of this study, we can conclude that MINST may represent a more effective approach than conventional therapy for controlling inflammatory markers in smokers who quit. Three months of smoking cessation could be a suitable timeframe to control smoking-induced elevated inflammatory mediators when combined with local therapy. Long-term follow-up durations are required to evaluate MINST outcomes in smokers who quit. Limitations of this study include the short follow-up period and the small sample size. Future studies should explore long-term outcomes and additional biomarkers.

## Conclusions

MINST, when combined with smoking cessation, effectively reduces salivary inflammatory biomarkers in smokers who quit. This approach shows promise for enhancing periodontal therapy outcomes.

## Data Availability

The original contributions presented in the study are included in the article/Supplementary Material, further inquiries can be directed to the corresponding author.
